# Cochlear Damage Affects Neurotransmitter Chemistry in the Central Auditory System

**DOI:** 10.3389/fneur.2014.00227

**Published:** 2014-11-19

**Authors:** Augustine C. Lee, Donald A. Godfrey

**Affiliations:** ^1^Department of Neurology, University of Toledo College of Medicine, Toledo, OH, USA; ^2^Division of Otolaryngology and Dentistry, Department of Surgery, University of Toledo College of Medicine, Toledo, OH, USA

**Keywords:** acetylcholine, aspartate, carboplatin, GABA, glutamate, glycine, taurine, tinnitus

## Abstract

Tinnitus, the perception of a monotonous sound not actually present in the environment, affects nearly 20% of the population of the United States. Although there has been great progress in tinnitus research over the past 25 years, the neurochemical basis of tinnitus is still poorly understood. We review current research about the effects of various types of cochlear damage on the neurotransmitter chemistry in the central auditory system and document evidence that different changes in this chemistry can underlie similar behaviorally measured tinnitus symptoms. Most available data have been obtained from rodents following cochlear damage produced by cochlear ablation, intense sound, or ototoxic drugs. Effects on neurotransmitter systems have been measured as changes in neurotransmitter level, synthesis, release, uptake, and receptors. In this review, magnitudes of changes are presented for neurotransmitter-related amino acids, acetylcholine, and serotonin. A variety of effects have been found in these studies that may be related to animal model, survival time, type and/or magnitude of cochlear damage, or methodology. The overall impression from the evidence presented is that any imbalance of neurotransmitter-related chemistry could disrupt auditory processing in such a way as to produce tinnitus.

## Introduction

### An emerging issue for society

Tinnitus, the perception of a monotonous sound, most commonly ringing (1), not actually present in the environment, can result from many different types of cochlear damage, including especially those resulting from acoustic trauma and ototoxic drugs (1, 2). It has been reported that over 10% of the US population suffers from hearing loss and nearly 20% from tinnitus specifically (3). Tinnitus and hearing loss were reported as the first and second most prevalent service-connected disabilities of all veterans and together constituted more than a third of the most prevalent disabilities of both all veterans and new veterans (4). Tinnitus has not been a major subject of study until recently. A general PubMed search for “tinnitus” reveals that at least 75% of all studies related to tinnitus have been published within the last 25 years. A need has been expressed for a better understanding of the rebalancing of excitatory and inhibitory signaling mechanisms in auditory disorders (5), but the study of the neurochemical basis for these signaling mechanisms is still in an early stage (2, 5).

### Purpose of this review

The vast majority of available data on the neurochemical changes in the central auditory system after cochlear damage is based on animal studies in rodents. These studies have used a variety of post-insult survival times. One study (6) found similar behaviorally measured tinnitus symptoms in chinchillas associated with three different patterns of cochlear damage following acoustic exposure, cisplatin, and carboplatin treatments. In our previous studies, we have found different effects on central auditory system neurotransmitter systems of different types of cochlear damage, including partial damage from acoustic trauma (7–10) and carboplatin treatment (11, 12), both of which have been associated with tinnitus (2, 13), and complete destruction via cochlear ablation (14–17). Cochlear ablation produces complete transection of auditory nerve fibers, which has been associated with tinnitus (18, 19). Although any chemical change could underlie a hearing disorder (5, 20–29), changes in neurotransmitter chemistry would affect the balance of interactions among neurons and could thereby lead to distorted hearing, which might be perceived as tinnitus. The purpose of this review is to compare the effects of various types of cochlear damage on the neurotransmitter chemistry in the central auditory system, thereby to document evidence that different changes in this chemistry can underlie similar behaviorally measured tinnitus symptoms. Although the types of cochlear damage employed in these studies can lead to tinnitus, behavioral evidence of tinnitus was not actually assessed in most of the studies.

### Categorization of review components

Most of the available data are summarized in tables. Effects of intense sound exposure, or acoustic trauma, and ototoxic drugs are compared to those of cochlear ablation. Most available data for ototoxic drugs are for carboplatin, but some data for salicylate, kanamycin, and neomycin are also included. Data from studies that used various survival times are grouped into short (1–2 weeks), mid (about 1 month), and long (2 months or more) times after the event leading to cochlear damage. Although short-term chemical changes could induce other systemic changes related to sustained hearing loss and tinnitus, the chemical changes related to chronic symptoms should presumably be present at long times after cochlear damage. Previous publications have expressed changes as increases, decreases, or no change (2, 5). We have taken a more objective approach of presenting the data from various quantitative studies numerically, as percent difference from control. Any change, no matter how small, could theoretically be important, particularly if consistent across neural regions; changes reported to be statistically significant are marked.

### Neurochemicals of interest

Most chemical data available for neurotransmitter systems after cochlear damage concern amino acids and acetylcholine. Of the amino acids, glutamate is well established as an excitatory neurotransmitter of auditory nerve fibers (30–35), and there is evidence that it is also a neurotransmitter of ascending (35), interneuronal (36), and descending (37, 38) pathways of the auditory system. There is some evidence for aspartate as a neurotransmitter of auditory nerve fibers (30–32, 35), but its association with the auditory nerve may also reflect its close metabolic relationship with glutamate (39, 40). Glutamine is also closely related metabolically to glutamate as an important precursor (40, 41), although predominantly located in glial cells (42). Both glycine and γ-aminobutyric acid (GABA) are well established as inhibitory neurotransmitters of the central auditory system, especially in the cochlear nucleus (CN), superior olive, and inferior colliculus (IC) (35, 43–55). Although taurine is not well established as an inhibitory neurotransmitter, there is evidence that taurine, in addition to its relatively high levels in glia (42, 56), is closely associated with GABAergic and glycinergic neurotransmission and may act as an agonist at GABA and glycine receptors (57–59). Available evidence suggests that acetylcholine serves as a neurotransmitter for several centrifugal pathways of the auditory system, particularly olivocochlear and olivo-CN connections (10, 35, 36, 60, 61). Its effects are mostly excitatory in the CN (62, 63) as well as other locations (64), and it may function as a neuromodulator as well as a neurotransmitter (64).

Box 1 **Table Notes**

Data are presented as percentage changes from control, which was usually an average for control or sham animals, but sometimes (as noted) from the corresponding contralateral structure of animals with unilateral damage. For IC, MG, and Aud Ctx, the affected side is contralateral to the damaged cochlea. Data from the different rodents are marked by C for chinchilla, G for guinea pig, H for hamster, and R for rat. The terms short, mid, and long refer to survival times after the cochlear damage: at or close to 1 week, about 1 month, and 2 months or more, respectively. Except as noted, data for level and synthesis were from quantitative assays of brain tissue. Data for amino acid levels after carboplatin in chinchilla IC, MG, and Aud Ctx and for acetylcholine synthesis in CN are unpublished data from one of us (DAG; treatment of animals was approved by and in accordance with existing regulations of the University of Toledo Health Science Campus Institutional Animal Care and Use Committee, which are consistent with guidelines of the National Institutes of Health). These data are less reliable since they were obtained from fewer animals, but they give some indication of chemical changes. Average data from mid and long survival times were combined for IC and compared to control chinchilla data.

^*^ Differences reported as statistically significant.
^†^ Godfrey et al. unpublished.
^‡^ Godfrey et al. in preparation.
^a^ Measured levels on lesioned side compared to contralateral.
^b^ Quantitative histochemical methods (90).
^c^ Where there was a decrease in sample dry weight per volume at long survival times, probably resulting from myelin loss, data were corrected for that change.
^d^ AMPA receptor binding.
^e^ CNQX (5,6-cyano-7-nitro-quinoxaline-2,3-dione) receptor binding; lesioned side compared to contralateral, superficial DCN is molecular layer only, and deep DCN is combined fusiform soma and deep layers.
^f^ Glutaminase activity 3 days after surgery, lesioned side compared to contralateral for total VCN, entered into PVCN space in table, and total DCN.
^g^ Vesicular glutamate transporter (VGLUT1) immunoreactivity.
^h^ Compared to contralateral.
^i^ Ratios of contralateral to ipsilateral values; data for glycine receptors based on immunoreactivity for the α1 subunit.
^j^ Data for IC total are protein levels measured with Western blot, and data for individual subdivisions of IC are optical density measures of immunoreactive somata; the more recent report (85) states that changes in GAD_65_ protein levels for total IC were predominantly in the membrane fraction.
^k^ The value for GABA receptors (muscimol binding to GABA_A_ receptors) in IC is for number of binding sites.
^l^ α1 subunit, GABRA1 immunoreactivity; the value for ventral IC was for the 10–16 kHz portion, which had the largest and only statistically significant effect.
^m^ For high characteristic frequency portion only; first value based on immunolabeled puncta counts and second based on Western blot; survival time 10 days (personal communication from Dr. S. Bao).
^n^ The value for GABA synthesis is for GAD_65_ level measured by Western blot; the value for receptors (GABA_A_) in IC is for number of binding sites; the value reported for combined dorsal and lateral IC was entered into the dorsal IC space in the table; subjects were treated with salicylate continuously for 4 months up to the time of euthanization.
^o^ Corrections were made for tissue shrinkage at longer times after cochlear damage.
^p^ Number of binding sites.
^q^ Immunolabeled puncta.
^r^ Choline acetyltransferase immunoreactivity.
^s^ Scopolamine binding to muscarinic receptors, lesioned side compared to contralateral.

Changes in neurotransmitter chemistry have been measured as changes in chemical level; synthetic capacity (synthesis), usually measured as enzyme activity; release, presumably from nerve terminals, by artificial stimulus; tissue uptake rate (uptake); and transmitter receptors (receptors), usually measured by receptor binding or immunohistochemistry. Although data for messenger RNA (mRNA) levels are available for some aspects of neurotransmitter chemistry (5, 52, 54) and often support the respective protein expression data, we did not include them in the tables of this review because there is often discordance between measurements of mRNA and protein expression (52). This implies that there are other complicating factors that may result in a lack of proportionality between mRNA expression and protein expression.

### Histological effects of cochlear damage

The most common methods of inducing cochlear damage in animal studies include cochlear ablation, ototoxic drugs, and intense sound (acoustic trauma). The different types of cochlear damage produce distinct histological effects in the central auditory system. Cochlear ablation leads to total degeneration of the auditory nerve (65, 66). Besides degeneration of auditory nerve fibers and terminals in the CN, there is also a hypertrophic reaction of nearby glial cells (67–69) and transneuronal effects in CN neurons and in neurons of higher auditory centers (66, 70). Cochlear ablation can also result in delayed, progressive volume decreases in heavily innervated portions of the CN (15, 16, 50). The auditory nerve degeneration following acoustic trauma (71–74) or carboplatin (75–77) is only partial. Nevertheless, transneuronal effects of acoustic trauma have been reported (73). Decreases in volume of CN regions after intense sound have been reported in some studies (28, 74) but not others (9). No volume changes in CN regions were found after carboplatin administration (11).

### Limitations of current data

Most data available for effects of cochlear damage on central auditory system chemistry have been obtained in rodents, including guinea pigs, rats, chinchillas, and hamsters. As with any comparison of data among animal species, interpretations are limited by interspecies differences. Another limitation of the data results from differences among individual animals within the same species. For this reason, comparisons between ipsilateral and contralateral sides in the same animal following a unilateral lesion are more reliable than comparisons between individual animals. However, any of the cochlear lesions can have bilateral effects, which can only be detected by comparisons to undamaged control animals.

## Notable Chemical Changes after Cochlear Damage

**Figure 1:**
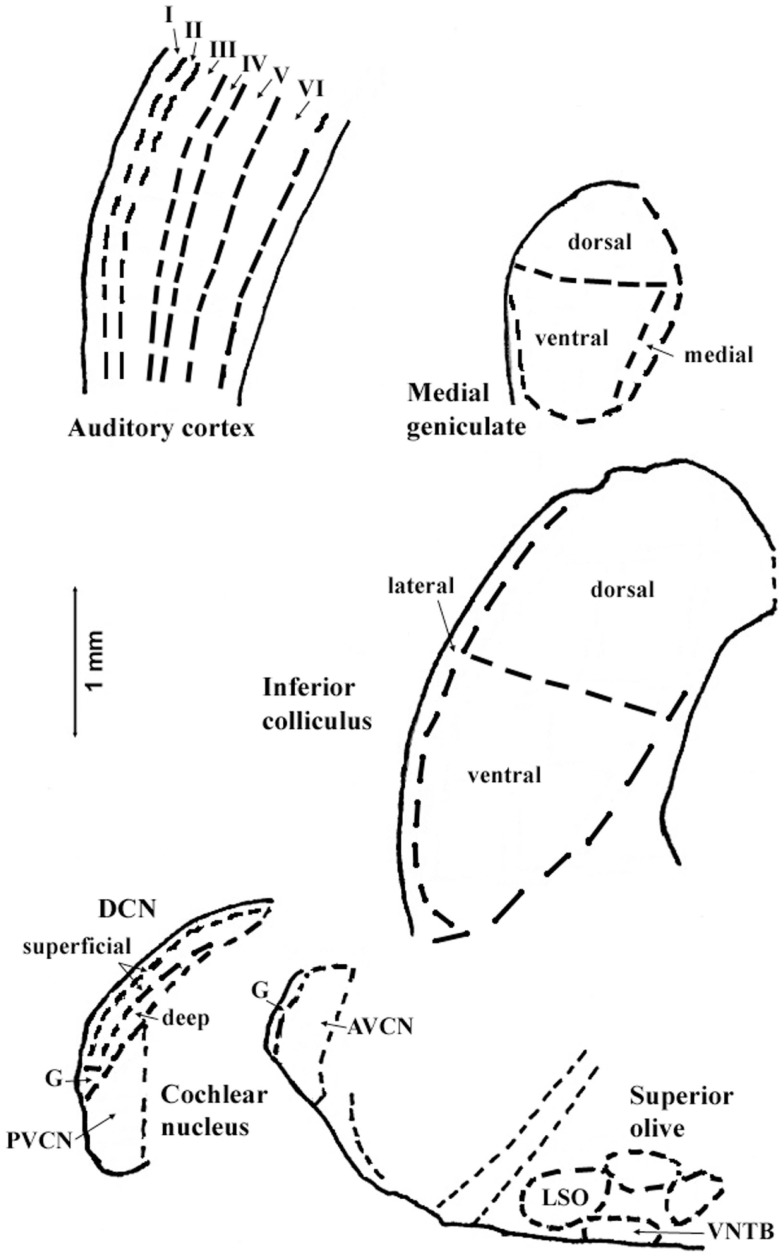
**Sections through central auditory regions for which data are shown in the tables**. Sections were traced from hamster brain. Names of subregions correspond to those in the tables. Abbreviations: I–VI, layers of auditory cortex; AVCN, anteroventral cochlear nucleus; DCN, dorsal cochlear nucleus; G, granular region; LSO, lateral superior olivary nucleus; PVCN, posteroventral cochlear nucleus; VNTB, ventral nucleus of the trapezoid body. Scale bar is shown at left; dorsal is up, and lateral is left.

The regions represented in the tables are identified in Figure 1. Each table contains available quantitative data for one neurochemical. In the following descriptions, which are keyed to Tables 1–7, we highlight the more prominent chemical changes or patterns presented in the tables. Each item in the tables includes a citation of its respective study.

**Table 1:** **Glutamate**.

Region	Measurement	Cochlear ablation			Intense sound			Carboplatin			Kanamycin
		Short	Mid	Long	Short	Mid	Long	Short	Mid	Long	Short
AVCN	Level	−27G* (31)^a^, −26R* (17)^b^, −16C*^,‡,b,c^	−18C*^,‡,b,c^, −13R (17)^b^	−23C*^,‡,b,c^, −8R (17)^b^			+7H (9)^b^	+5C (12)^b^	−2C (12)^b^	−22C (12)^b^	
	Synthesis								−30C* (11)^b^		−63G* (38)^g^
	Release	−52G* (78)	−38G* (78)	−19G (78)	+72C* (79)^d^		+6C (79)^d^				
	Uptake	−31G* (78)	−43G* (78)	−40G (78)	−60C* (79)^d^		−47C* (79)^d^				
	Receptors	−35G* (80)^d^, −25R* (81)^e^	−15R (81)^e^, −4G (80)^d^	+6G (80)^d^	0C (79)^d^		+70C* (79)^d^				

PVCN	Level	−32R* (17)^b^, −27G* (31)^a^, −20C* (16)^‡,b,c^	−66C* (16)^‡,b,c^, −36R* (17)^b^	−59C* (16)^‡,b,c^, −25R* (17)^b^	−7H (8)^b^		+15H* (9)^b^	0C (12)^b^	−6C (12)^b^	−39C* (12)^b^	
	Synthesis	−28G* (33)^f^							−38C* (11)^b^		−63G* (38)^g^
	Release	−76G* (78)	−13G (78)	−8G (78)	+131C* (79)^d^		+30C (79)^d^				
	Uptake	−43G* (78)	−50G* (78)	−17G (78)	−47C* (79)^d^		−50C* (79)^d^				
	Receptors	−14R* (81)^e^, +83G* (80)^d^	−14R (81)^e^, −10G (80)^d^	−13G (80)^d^	−5C (79)^d^		+62C* (79)^d^				

DCN total	Synthesis	−2G (33)^f^									
	Release	−50G* (78)	−53G* (78)	−52G* (78)	+72C* (79)^d^		+44C (79)^d^				
	Uptake	−38G* (78)	−25G (78)	+21G (78)	−41C* (79)^d^		−12C (79)^d^				

DCN deep	Level	−19R (17)^b^, −18C^‡,b,c^, −6G (31)^a^	−22C^‡,b,c^, −15R (17)^b^	−21C^‡,b,c^, −14R (17)^b^	−16H* (8)^b^	−9H (8)^b^	+16H (9)^b^	+8C (12)^b^	−1C (12)^b^	−6C (12)^b^	
	Synthesis								−5C (11)^b^		+13G (38)^g^
	Receptors	−18R* (81)^e^, +60G* (80)^d^	−17R (81)^e^, 0G (80)^d^	+5G (80)^d^							

DCN superficial	Level	−10C^‡,b,c^, −6R (17)^b^, +7G (31)^a^	−9R (17)^b^, −2C^‡,b,c^	−8C^‡,b,c^, −3R (17)^b^	−13H* (8)^b^	−3H (8)^b^	+6H (9)^b^	+3C (12)^b^	−5C (12)^b^	0C (12)^b^	
	Synthesis								−8C (11)^b^		0G (38)^g^
	Receptors	+8R (81)^e^, +10G (80)^d^	−7G (80)^d^, +3R (81)^e^	−5G (80)^d^	0C (79)^d^		−10C (79)^d^				

CN granular	Level	−18R (17)^b^, −16G (31)^a^, −14C^‡,b,c^	−3R (17)^b^, +8C^‡,b,c^	−12R (17)^b^, 0C^‡,b,c^			+5H (9)^b^		−7C (11)^b^		
	Synthesis								−8C (11)^b^		+7G (38)^g^
	Receptors	−2G (80)^d^	−24G* (80)^d^	+9G (80)^d^							

LSO	Level	+16R (17)^b^	+10R (17)^b^	−24R (17)^b^							
	Release	−20G (78)	+1G (78)	+18G* (78)							
	Uptake	−18G* (78)	−1G (78)	+34G* (78)							
	Receptors	−39G* (80)^d^	−9G (80)^d^	−23G* (80)^d^							

IC dorsal	Level						+12H* (9)^b^		−3C (31^a^, 78)^†,b^	
	Receptors	+6G (80)^d^	−4G (80)^d^	+2G (80)^d^						

IC ventral	Level						+10H* (9)^b^		+15C^†,b^	
	Release	+14G (78)	+55G* (78)	+41G* (78)							
	Uptake	−14G (78)	−5G (78)	−5G (78)							
	Receptors	+12G (80)^d^	+18G (80)^d^	+6G (80)^d^							

IC lateral	Level						+7H (9)^b^		+16C^†,b^	
	Receptors	+16G (80)^d^	−2G (80)^d^	−9G* (80)^d^						

MG total	Level							+7C^†,b^	+7C^†,b^	+1C^†,b^	
MG dorsal	Level						+6H (9)^b^				
MG ventral	Level						+2H (9)^b^				
MG medial	Level						−2H (9)^b^				
Aud Ctx total	Level							+5C^†,b^	+4C^†,b^	−8C^†,b^	
Aud Ctx layer I	Level						+6H (9)^b^				
Aud Ctx layer II	Level						+6H (9)^b^				
Aud Ctx layer III	Level						+6H (9)^b^				
Aud Ctx layer IV	Level						+8H (9)^b^				
Aud Ctx layer V	Level						+8H (9)^b^				
Aud Ctx layer VI	Level						+8H (9)^b^				

*See **Box 1** for table notes*.

### Glutamate

**Figure 2:**
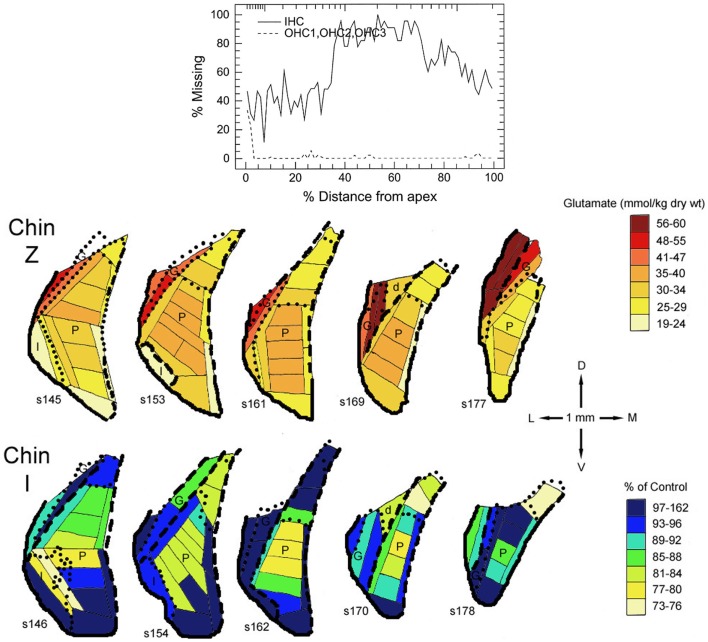
**Maps of glutamate levels in the posteroventral cochlear nucleus (PVCN) of a control chinchilla (Chin Z) and of a chinchilla treated 29 days earlier with 100 mg/kg carboplatin by intraperitoneal injection (Chin I)**. The loss of cochlear inner (IHC) and outer (OHC) hair cells is plotted in the diagram at the top vs. distance from the cochlear apex. Glutamate levels are color coded for Chin Z. For Chin I, the glutamate levels are expressed as percentages of those for comparable locations in Chin Z. For detailed methods, see Godfrey et al. (12).

In all three species studied, cochlear ablation resulted in decreased glutamate levels in each time category and in all regions receiving sizable innervation from auditory nerve fibers (AVCN, PVCN, and deep DCN, Table 1). Effects in superficial DCN and granular regions were smaller and inconsistent. Glutamate release was also consistently decreased at all times. Uptake was decreased in each time category of the CN regions except in the DCN at long survival times. Changes in glutamate receptors (AMPA type) were not consistent in guinea pig, but in rat they were all decreased in regions receiving sizable innervation from auditory nerve fibers. Large decreases in glutamate transporter (synthesis) in VCN (AVCN + PVCN) following kanamycin damage in guinea pigs correlated with decreased glutamate levels after cochlear ablation. Glutamate level and synthesis data in VCN after carboplatin resembled those for cochlear ablation except for a slower progression. To some extent, this slower progression paralleled a slower progression of the cochlear damage following carboplatin administration (12). At the mid time, inner hair cell loss was partial. In one chinchilla, where the loss of inner hair cells was largest in an intermediate portion of the cochlear spiral, the decrease in glutamate level was larger in an intermediate portion of the PVCN than in more dorsal or ventral locations (Figure 2). The effects of intense sound were more complex. In contrast to the data for other types of cochlear damage, glutamate levels increased in all CN regions at long times after intense sound. Although glutamate uptake decreased, as with cochlear ablation, release increased at all measured survival times. Receptors in the chinchilla VCN increased greatly at longer times after intense sound. A non-quantitative immunohistochemical study (not included in Table 1) reported a redistribution of *N*-methyl-d-aspartate (NMDA) type glutamate receptors, from mostly axo-somatic to mostly axo-dendritic locations, in the VCN at various times up to more than a year after cochlear ablation (91). Another immunohistochemical study (not included in Table 1 because non-quantitative) found a loss of vesicular glutamate transporter (vGLUT1) from auditory nerve terminals and its appearance in VCN neuron somata 3 days after mechanical ablation of cochlear hair cells (92).

For more central auditory regions after cochlear ablation or carboplatin, many changes for glutamate were increases, unlike corresponding effects in the CN. As in the CN, glutamate levels increased in almost all central auditory regions at long times after intense sound.

Decreases in glutamate chemistry in the CN coupled with degeneration of auditory nerve fibers are consistent with evidence that glutamate serves as transmitter of auditory nerve fibers (30–35). The mixed neurochemical changes of glutamate in the more central auditory regions after cochlear ablation or ototoxic damage may correlate with the evidence that glutamate serves as a neurotransmitter of various ascending (35), interneuronal (36), and descending (37, 38) pathways. These pathways would undergo relatively little physical degeneration after cochlear damage but may undergo complex compensatory changes. On the other hand, the effects on glutamate neurotransmission in the central auditory system at long times after intense sound differ from those of cochlear ablation and carboplatin administration. Under these conditions, glutamate is present at higher levels and is released more efficiently to receptors that are more sensitive; whereas glutamate is removed (uptake) from receptors less efficiently. All these changes together would make the central auditory system more excitable, which could result in or contribute to tinnitus.

### Aspartate

Results for aspartate levels after cochlear ablation resemble those for glutamate levels with one notable, unexpected exception: in chinchillas, aspartate levels increased at short survival times (Table 2). Decreases in aspartate level in the CN after carboplatin treatment were similar to those for glutamate except that they developed more rapidly. At long survival times after intense sound, aspartate levels were consistently and often significantly increased, more so than glutamate levels, in all central auditory regions. Thus, for aspartate, even more so than for glutamate, the changes at long times after intense sound were opposite to those after cochlear ablation or carboplatin.

**Table 2:** **Aspartate**.

Region	Measurement	Cochlear ablation			Intense sound			Carboplatin		
		Short	Middle	Long	Short	Mid	Long	Short	Mid	Long
AVCN	Level	−42G* (31)^a^, −19R* (17)^b^, +13C^‡,b,c^	−19C*^,‡,b,c^, 0R (17)^b^	−18R (17)^b^, −12C^‡,b,c^			+17H* (9)^b^	−20C (12)^b^	−27C* (12)^b^	−28C* (12)^b^
PVCN	Level	−37G* (31)^a^, −31R* (17)^b^, +58C*^,‡,b,c^	−63C*^,‡,b,c^, −31R* (17)^b^	−57C*^,‡,b,c^, −18R (17)^b^	−11H (8)^b^		+36*H (9)^b^	−14C (12)^b^	−24C (12)^b^	−40C* (12)^b^
DCN deep	Level	−16R (17)^b^, −7G (31)^a^, +19C^‡,b,c^	−30C^‡,b,c^, +3R (17)^b^	−24C^‡,b,c^, −4R (17)^b^	−3H (8)^b^	−30H (8)^b^	+21H (9)^b^	−19C (12)^b^	−9C (12)^b^	−19C* (12)^b^
DCN superficial	Level	−9C^‡,b,c^, −5R (17)^b^, 0G (31)^a^	−8C^‡,b,c^, −3R (17)^b^	−1R (17)^b^, +2C^‡,b,c^	−8H (8)^b^	−13H (8)^b^	+11H (9)^b^	−6C (12)^b^	−2C (12)^b^	−10C (12)^b^
CN granular	Level	−10G (31)^a^, −7C^‡,b,c^, +2R (17)^b^	−6C^‡,b,c^, +9R (17)^b^	−13R (17)^b^, −2C^‡,b,c^			+23H* (9)^b^		−4C (11)^b^	

LSO	Level	+18R (17)^b^	+4R (17)^b^	−39R (17)^b^						

IC dorsal	Level						+12H* (9)^b^		+9C^†,b^
IC ventral	Level						+10H* (9)^b^		−1C^†,b^
IC lateral	Level						+12H* (9)^b^		+31C^†,b^

MG total	Level							−16C^†,b^	−14C^†,b^	−22C^†,b^
MG dorsal	Level						+13H* (9)^b^			
MG ventral	Level						+12H* (9)^b^			
MG medial	Level						+12H* (9)^b^			

Aud Ctx total	Level							−7C^†,b^	+2C^†,b^	−25C*^,†,b^
Aud Ctx layer I	Level						+12H (9)^b^			
Aud Ctx layer II	Level						+13H (9)^b^			
Aud Ctx layer III	Level						+10H (9)^b^			
Aud Ctx layer IV	Level						+7H (9)^b^			
Aud Ctx layer V	Level						+16H* (9)^b^			
Aud Ctx layer VI	Level						+21H* (9)^b^			

*See **Box 1** for table notes*.

Parallel changes of aspartate neurochemistry are consistent with its close metabolic relationship with glutamate (39, 40). Increases in levels of aspartate at long times after intense sound exposure, combined with its ease of conversion to glutamate through the activity of aspartate aminotransferase (40), would contribute to a greater excitability of the central auditory system.

### Glutamine

Although glutamine is a major precursor for synthesis of glutamate, its changes after cochlear damage, except in the lateral superior olivary nucleus (LSO), were usually opposite to those of glutamate (Table 3). Intense sound damage resulted in no clear effect on glutamine levels. The most consistent effects were increased glutamine levels in VCN and deep DCN of rat after cochlear ablation and of chinchilla after carboplatin administration. Since glutamine levels are relatively high in glial cells (42, 56), these increases could reflect glial hypertrophy in the regions where auditory nerve fibers are degenerating.

**Table 3:** **Glutamine**.

Region	Measurement	Cochlear ablation			Intense sound			Carboplatin		
		Short	Mid	Long	Short	Mid	Long	Short	Mid	Long
AVCN	Level	−9C*^,‡,b,c^, −6R (17)^b^	+4C^‡,b,c^, +6R (17)^b^	+12R (17)^b^, +16C*^,‡,b,c^			−2H (9)^b^	+6C (12)^b^	+14C (12)^b^	+16C (12)^b^
PVCN	Level	−7C^‡,b,c^, +2R (17)^b^	−22C*^,‡,b,c^, +11R (17)^b^	+1C^‡,b,c^, +35R* (17)^b^	−3H (8)^b^		+3H (9)^b^	+17C (12)^b^	+11C (12)^b^	+3C (12)^b^
DCN deep	Level	−19C*^,‡,b,c^, +2R (17)^b^	−8C^‡,b,c^, +16R* (17)^b^	−12C^‡,b,c^, +21R* (17)^b^	+11H (8)^b^	−23H (8)^b^	0H (9)^b^	+19C (12)^b^	+11C (12)^b^	+15C (12)^b^
DCN superficial	Level	−15C*^,‡,b,c^, −5R (17)^b^	0C^,‡,b,c^, +5R (17)^b^	−7R (17)^b^, −6C^‡,b,c^	+6H (8)^b^	−14H (8)^b^	−2H (9)^b^	+12C (12)^b^	+1C (12)^b^	+5C (12)^b^
CN granular	Level	−18C*^‡,b,c^, −3R (17)^b^	+7R (17)^b^, +10C^‡,b,c^	0C^‡,b,c^, 0R (17)^b^			−3H (9)^b^		−18C* (11)^b^	

LSO	Level	+17R (17)^b^	+4R (17)^b^	−12R (17)^b^						

IC dorsal	Level						+2H (9)^b^		−4C^†,b^
IC ventral	Level						+3H (9)^b^		+16C^†,b^
IC lateral	Level						+3H (9)^b^		−5C^†,b^

MG total	Level							−5C^†,b^	−12C^†,b^	−8C^†,b^
MG dorsal	Level						0H (9)^b^			
MG ventral	Level						−4H (9)^b^			
MG medial	Level						−3H (9)^b^			

Aud Ctx total	Level							−15C*^,†,b^	−6C^†,b^	−10C^†,b^
Aud Ctx layer I	Level						+5H (9)^b^			
Aud Ctx layer II	Level						+5H (9)^b^			
Aud Ctx layer III	Level						+1H (9)^b^			
Aud Ctx layer IV	Level						+4H (9)^b^			
Aud Ctx layer V	Level						+5H (9)^b^			
Aud Ctx layer VI	Level						+1H (9)^b^			

*See **Box 1** for table notes*.

### γ-Aminobutyric acid

After cochlear ablation, there were striking decreases of GABA levels in chinchilla VCN [which were bilateral (16)], especially at shorter times (Table 4). In rat, there were [bilateral (17)] decreases in AVCN but not in PVCN. There were similar but less striking decreases in chinchilla VCN GABA levels after carboplatin (12). In superficial DCN, which receives little innervation from auditory nerve fibers (93, 94), GABA levels surprisingly decreased in rat after cochlear ablation and in chinchilla after carboplatin administration. One possible explanation for these decreases in GABA levels could be non-specific effects of trauma (95), which would be produced by cochlear damage, since GABA is not substantially related to auditory nerve fibers (17). A transneuronal effect of cochlear ablation on GABA levels in ipsilateral CN neurons is supported by an immunohistochemical study in rats (96). Counts of GABA-immunoreactive neurons decreased by 56% at 1 week and by 63% at 2 weeks after cochlear ablation, compared to contralateral, but there was no significant difference between the sides at 1 month (these data were not included in Table 4 due to uncertainty of sampling location within the CN).

**Table 4:** **GABA**.

Region	Measurement	Cochlear ablation			Intense sound			Carboplatin			Neomycin	Salicylate
		Short	Mid	Long	Short	Mid	Long	Short	Mid	Long	Mid	Long
CN total	Synthesis	−18G* (44)^h^										

AVCN	Level	−81C*^‡,b,c^, −14R (17)^b^	−17R (17)^b^, −8C^‡,b,c^	−15R (17)^b^, −14C^‡,b,c^			+2H (9)^b^	−5C (12)^b^	−13C (12)^b^	−18C (12)^b^		
	Release	+8G (51)										
	Uptake	+20G (51)										

PVCN	Level	−41C*^,‡,b,c^, +5R (17)^b^	−42C*^‡,b,c^, −7R (17)^b^	−26C^‡,b,c^, +19R* (17)^b^	−7H (8)^b^		+3H (9)^b^	−18C* (12)^b^	−22C* (12)^b^	−18C* (12)^b^		
	Release	+5G (51)										
	Uptake	+9G (51)										

DCN total	Release	−2G (51)										
	Uptake	0G (51)										
DCN deep	Level	−15C^‡,b,c^, 0R (17)^b^	−6C^‡,b,c^, 0R (17)^b^	−15C^‡,b,c^, +11R (17)^b^	+3H (8)^b^	+14H (8)^b^	0H (9)^b^	+22C (12)^b^	−1C (12)^b^	+11C (12)^b^		
DCN superficial	Level	−17R (17)^b^, +2C^‡,b,c^	−20R* (17)^b^, −4C^‡,b,c^	−10R (17)^b^, −8C^‡,b,c^	0H (8)^b^	−3H (8)^b^	+1H (9)^b^	−1C (12)^b^	−14C (12)^b^	−20C* (12)^b^		

CN granular	Level	−6C^‡,b,c^, −4R (17)^b^	−13R (17)^b^, +26C^‡,b,c^	−5R (17)^b^, +2C^‡,b,c^			−2H (9)^b^		−24C* (11)^b^			

LSO	Level	−42R* (17)^b^	−48R* (17^b^)	−47R* (17)^b^								

IC total	Synthesis	−34R* (82)^i^, −7G* (44)^h^		−30R* (82)^i^	+9R (83)^j^	−21R (83)^j^						+57R* (84)^n^
	Receptors				−1R (85)^k^	+19R* (85)^k^						−17R* (84)^n^
IC dorsal	Level						+9H (9)^b^		−20C^†,b^		
	Synthesis				−13R (83)^j^	−4R (83)^j^						
	Receptors				−15G (55)^l^, 0R (85)^k^	+24R* (85)^k^						−15R (84)^n^
IC ventral	Level						+11H* (9)^b^		+15C^†,b^		
	Synthesis				−11R (83)^j^	−2R (83)^j^						
	Release	+26G* (51)	+11G (51)	+39G* (51)							−74G (86)	
	Uptake	+6G (51)	−5G (51)	−29G* (51)								
	Receptors				−36G* (55)^l^, −2R (85)^k^	+22R (85)^k^						−22R* (84)^n^
IC lateral	Level						+11H* (9)^b^		−15C^†,b^			
	Synthesis				−12R (83)^j^	+3R (83)^j^						
	Receptors				−13G (55)^l^, +9R (85)^k^	+20R (85)^k^						

MG total	level							−13C^†,b^	−22C*^,†,b^	−15C^†,b^		
MG dorsal	Level						−6H (9)^b^					
MG ventral	Level						+2H (9)^b^					
MG medial	Level						+12H (9)^b^					
Aud Ctx total	Level							−22C^†,b^	−14C^†,b^	−20C^†,b^		
	Synthesis				−57R* (87)^m^, −32R* (87)							
Aud Ctx layer I	Level						+1H (9)^b^					
Aud Ctx layer II	Level						+1H (9)^b^					
Aud Ctx layer III	Level						+6H (9)^b^					
Aud Ctx layer IV	Level						+2H (9)^b^					
Aud Ctx layer V	Level						+7H (9)^b^					
Aud Ctx layer VI	Level						+2H (9)^b^					

*See **Box 1** for table notes*.

There were also consistent decreases of GABA level in the rat LSO ipsilateral to cochlear ablation, which might result from a retrograde effect on olivocochlear neurons after destruction of their terminals (17). Measurements for GABA, especially GABA receptors, usually showed decreases after ototoxic drug administration. The striking increase in GABA synthetic capacity in the IC after continuous, long-term salicylate administration in rats (84) might represent a compensatory response to the decrease in GABA receptors. After cochlear ablation, GABA synthesis and uptake decreased in the IC, whereas its release increased. These directions of change for release and uptake were similar to those for glutamate. In another study (not included in Table 4 due to uncertainty of sampling location within the IC), counts of GABA-immunoreactive neurons decreased by 33% in the contralateral IC at 1 week after unilateral cochlear ablation, but the decrease was not statistically significant at 1 month (97). Although GABA receptors and synthesis usually decreased in the IC at short times after intense sound, GABA receptors and levels increased at mid and long times, respectively. Similarly, GABA levels showed some tendency to increase in the medial geniculate (MG) and auditory cortex (Aud Ctx) at long times after intense sound.

Overall, the changes in the neurochemistry of GABA after cochlear damage do not consistently support a simultaneous correlation between loss of GABA inhibition and tinnitus.

### Glycine

In the central auditory system, up through the IC, glycine receptors were almost always decreased at all times after cochlear ablation (Table 5). There are some striking contrasts, such as that between increased uptake and decreased release in AVCN and DCN total, and that between decreased levels in chinchilla PVCN and increased levels in rat PVCN. In both deep and superficial portions of the DCN, glycine levels decreased slightly after cochlear ablation but increased after carboplatin administration. These directions of change resembled those for glutamine in chinchillas but were opposite to those for glutamate and aspartate. At short times after neomycin administration, large decreases in density of glycine-immunoreactive puncta in the CN were localized on specific neuron types including spherical bushy cells, globular bushy cells, and radiate cells in the VCN and fusiform cells in the DCN (88). These decreases in numbers of glycine-immunoreactive puncta in rat CN (88), and also superior olive (89), were much more striking than decreases in measured glycine levels in the same regions after cochlear ablation. This suggests compensating increases of glycine levels in some structures besides puncta, such as in reacting glial cells (67–69). In cerebellar cultures, glial cells have been reported to contain higher glycine levels than neurons (98). The only major change in glycine chemistry after intense sound was a prominent decrease of receptors in DCN total (52). In this same study, immunohistochemistry for glycine receptor subunits suggested that these decreases were most prominent at fusiform cells. Thus, the increased spontaneous activity of fusiform cells after intense sound exposure, which has been associated with tinnitus (99, 100), may at least partially result from decreased inhibitory input because of less glycine receptors (52) and maybe also less glycine neurotransmitter levels (9).

**Table 5:** **Glycine**.

Region	Measurement	Cochlear ablation			Intense sound			Carboplatin			Neomycin	
		Short	Mid	Long	Short	Mid	Long	Short	Mid	Long	Short	Mid
AVCN	Level	−11C^‡,b,c^, +2R (17)^b^	−3C^‡,b,c^, −1R (17)^b^	+5C^‡,b,c^, −4R (17)^b^			−5H (9)^b^	+13C (12)^b^	+5C (12)^b^	−2C (12)^b^	−58R* (88)^q^	
	Release	+3G (51)	−34G* (51)	−5G (51)								
	Uptake	−3G (51)	+21G (51)	+53G* (51)								
	Receptors	−24G* (50)^o^	−8G (50)^o^	−11G (50)^o^								

PVCN	Level	−35C*^‡,b,c^, +10R (17)^b^	−40C*^‡,b,c^, +8R (17)^b^	−11C^‡,b,c^, +29R* (17)^b^	−1H (8)^b^		−1H (9)^b^	−3C (12)^b^	−10C (12)^b^	−7C (12)^b^	−54R* (88)^q^	
	Release	−4G (51)	+16G (51)	+8G (51)								
	Uptake	+5G (51)	+10G (51)	+79G* (51)								
	Receptors	−8G (50)^o^	+3G (50)^o^	−29G* (50)^o^								

DCN total	Release	−5G (51)	−42G* (51)	−55G* (51)								
	Uptake	0G (51)	+47G* (51)	+55G* (51)								
	Receptors						−44R* (52)^p^					
DCN deep	Level	−10C^‡,b,c^, −5R (17)^b^	−11C^‡,b,c^, −1R (17)^b^	−12C^‡,b,c^, −2R (17)^b^	−2H (8)^b^	−17H (8)^b^	0H (9)^b^	+43C* (12)^b^	+22C (12)^b^	+17C (12)^b^		
	Receptors	+2G (50)^o^	+16G* (50)^o^	0G (50)^o^								
DCN superficial	Level	−6R (17)^b^, +1C^‡,b,c^	−9C^‡,b,c^, −8R (17)^b^	−6C^‡,b,c^, −3R (17)^b^	−5H (8)^b^	−13H (8)^b^	−8H (9)^b^	+22C (12)^b^	+23C (12)^b^	+1C (12)^b^	−63R* (88)^q^	
	Receptors	−6G (50)^o^	−10G* (50)^o^	−11G* (50)^o^								

CN granular	Level	−14C^‡,b,c^, +23R (17)^b^	+7C^‡,b,c^, +16R (17)^b^	−10R (17)^b^, +6C^‡,b,c^			−11H (9)^b^		−23C* (11)^b^			
	Receptors	−3G (50)^o^	0G (50)^o^	−12G* (50)^o^								

LSO	Level	−13R (17)^b^	+2R (17)^b^	−2R (17)^b^							−24R* (89)^q^	
	Release	0G (51)	−5G (51)	−3G (51)								
	Uptake	−26G* (51)	0G (51)	+7G (51)								
	Receptors	−22G* (50)^o^	−29G* (50)^o^	−55G* (50)^o^								

VNTB	Level										−46R* (89)^q^	

IC total	Receptors	−22R* (82)^i^		−39R* (82)^i^								
IC dorsal	Level						−3H (9)^b^		+11C^†,b^		
	Receptors	−10G (50)^o^	+1G (50)^o^	−10G (50)^o^								
IC ventral	Level						+6H (9)^b^		+12C^†,b^		
	Release											−9G (86)
	Receptors	−14G* (50)^o^	0G (50)^o^	−14G* (50)^o^								
IC lateral	Level						1H (9)^b^		+1C^†,b^		
	Receptors	+7G (50)^o^	−9G (50)^o^	−9G (50)^o^								

MG total	Level							+10C^†,b^	−12C^†,b^	+11C^†,b^		
MG dorsal	Level						−8H (9)^b^					
MG ventral	Level						−8H (9)^b^					
MG medial	Level						−9H (9)^b^					

Aud Ctx total	Level							−10C^†,b^	−13C^†,b^	−4C^†,b^		
Aud Ctx layer I	Level						+1H (9)^b^					
Aud Ctx layer II	Level						−5H (9)^b^					
Aud Ctx layer III	Level						+3H (9)^b^					
Aud Ctx layer IV	Level						+9H (9)^b^					
Aud Ctx layer V	Level						−2H (9)^b^					
Aud Ctx layer VI	Level						0H (9)^b^					

*See **Box 1** for table notes*.

### Taurine

In regions of the CN that are well innervated by auditory nerve fibers, taurine levels were consistently increased in rat after cochlear ablation, but effects in chinchilla were mixed (Table 6). After carboplatin administration, there were increased taurine levels in all regions of the chinchilla CN except the granular region (11, 12) and consistent increases in more central regions. Increases of taurine after cochlear damage in regions densely innervated by the auditory nerve could, as with glutamine, be related to glial hypertrophy in regions where auditory nerve fibers are degenerating, since taurine concentrations are relatively high in glia (42, 56), but this would not account for increases in more central auditory regions. Intense sound led at long times to slight-to-moderate decreases of taurine levels in all regions of the hamster central auditory system (8, 9). These changes in taurine levels were almost always opposite to those for aspartate. Because of taurine’s association with GABA and glycine neurotransmission (57–59), decreased taurine levels could be associated with decreased inhibitory activity in the central auditory system, which could result in tinnitus. Previous animal studies have found that a decreased blood taurine concentration is associated with hearing loss (101) and that taurine administration can decrease behavioral evidence of tinnitus (102).

**Table 6:** **Taurine**.

Region	Measurement	Cochlear ablation			Intense sound			Carboplatin		
		Short	Mid	Long	Short	Mid	Long	Short	Mid	Long
AVCN	Level	−24C*^‡,b,c^, +14R (17)^b^	+12C^‡,b,c^, +14R* (17)^b^	+14C^‡,b,c^, +25R* (17)^b^			−9H (9)^b^	+17C (12)^b^	+19C (12)^b^	+34C (12)^b^
PVCN	Level	−53C*^‡,b,c^, +5R (17)^b^	−53C*^‡,b,c^, +23R (17)^b^	−10C^‡,b,c^, +61R* (17)^b^	−7H (8)^b^		−13H* (9)^b^	−10C (12)^b^	+14C (12)^b^	+25C (12)^b^
DCN deep	Level	−12C^‡,b,c^, +1R (17)^b^	−4C^‡,b,c^, +9R (17)^b^	−10C^‡,b,c^, +24R (17)^b^	−11H (8)^b^	−21H* (8)^b^	−10H (9)^b^	+3C (12)^b^	+13C (12)^b^	+25C* (12)^b^
DCN superficial	Level	−12R (17)^b^, 0C^‡,b,c^	−6R (17)^b^, +3C^‡,b,c^	+3C^‡,b,c^, +8R (17)^b^	−12H* (8)^b^	−21H (8)^b^	−7H (9)^b^	+2C (12)^b^	+9C (12)^b^	+7C (12)^b^
CN granular	Level	−21C^‡,b,c^, +4R (17)^b^	−1R (17)^b^, +13C^‡,b,c^	−12R (17)^b^, +1C^‡,b,c^			−10H (9)^b^		−7C (11)^b^	

LSO	Level	−6R (17)^b^	0R (17)^b^	−2R (17)^b^						

IC dorsal	Level						−12H* (9)^b^		+17C^†,b^
IC ventral	Level						−6H* (9)^b^		+34C^†,b^
IC lateral	Level						−6H (9)^b^		+31C^†,b^

MG total	Level							+26C*^,†,b^	+21C^†,b^	+41C*^†,b^
MG dorsal	Level						−11H (9)^b^			
MG ventral	Level						−13H* (9)^b^			
MG medial	Level						−4H (9)^b^			

Aud Ctx total	Level							+44C*^,†,b^	+46C*^,†,b^	+59C*^,†,b^
Aud Ctx layer I	Level						−3H (9)^b^			
Aud Ctx layer II	Level						−4H (9)^b^			
Aud Ctx layer III	Level						−4H (9)^b^			
Aud Ctx layer IV	Level						−5H (9)^b^			
Aud Ctx layer V	Level						−6H (9)^b^			
Aud Ctx layer VI	Level						−9H (9)^b^			

*See **Box 1** for table notes*.

### Acetylcholine

In the CN after cochlear ablation, the synthetic capacity for acetylcholine (choline acetyltransferase activity) increased at mid times, but it returned toward control levels in most regions at long times (Table 7). Muscarinic acetylcholine receptors increased in CN regions at mid and long times after cochlear ablation. Synthetic capacity for acetylcholine increased in all CN regions through 2 months after intense sound, but the increase was not maintained through 5 months except in the granular region. Some of these changes may correlate with formation of new synapses after acoustic trauma (73). The sustained increase of choline acetyltransferase activity in the CN granular region (7, 10), as well as an increase in muscarinic acetylcholine receptor sensitivity in the superficial DCN (103), could be consistent with formation of new cholinergic synapses or upregulation of existing cholinergic synapses upon granule cells. Since many granule cells form excitatory glutamatergic synapses with fusiform and cartwheel cells in the superficial DCN (36, 104), increased cholinergic activity, leading to increased granule cell activity, could change the balance of excitatory and inhibitory input to DCN fusiform cells and thereby alter their activity (10, 105–107). Increased spontaneous activity of DCN fusiform cells after acoustic trauma has been associated with tinnitus (99, 100).

**Table 7:** **Acetylcholine**.

Region	Measurement	Cochlear ablation			Intense sound			Carboplatin		
		Short	Mid	Long	Short	Mid	Long	Short	Mid	Long
AVCN	Synthesis	+19R (14)^b^ and +91R* (60)^r^	+54R* (14)^b^	−4R (14)^b^	+252H* (7)^b^		+28H (7^b^) and −23H (10)^b^			
	Receptors	+6R (15)^s^	+28R* (15)^s^	+67R* (15)^s^						
PVCN	Synthesis	+37R* (14)^b^	+54R* (14)^b^	+40R* (14)^b^	+27H (7)^b^		+27H (7)^b^		−32C^†,b^
	Receptors	−15R* (15)^s^	+18R (15)^s^	+70R*(15)^s^						
DCN deep	Synthesis	+8R (14)^b^	+25R (14)^b^	+7R (14)^b^	+37H (7)^b^		+36H (7)^b^ and −21H (10)^b^		+9C^†,b^
	Receptors	+7R (15)^s^	+20R*(15)^s^	+22R*(15)^s^						
DCN superficial	Synthesis	−3R (14)^b^	+15R (14)^b^	−9R (14)^b^	+35H (7)^b^		+16H (7^b^) and −11H (10)^b^		+62C^†,b^
	Receptors	+3R (15)^s^	+8R (15)^s^	+11R*(15)^s^						
CN granular	Synthesis	−24R (14)^b^	+49R* (14)^b^	−5R (14)^b^	+38H* (7)^b^		+28H (7)^b^ and +38H* (10)^b^		−46C^†,b^
	Receptors	+1R (15)^s^	+11R*(15)^s^	+27R*(15)^s^						

LSO	Synthesis	−25R* (14)^b^	−5R (14)^b^	−37R* (14)^b^			+40H* (10)^b^			
VNTB	Synthesis	+19R (14)^b^	−19R (14)^b^	−22R* (14)^b^			+32H (10)^b^			

IC dorsal	Synthesis						+10H (10)^b^			
IC ventral	Synthesis						−12H (10)^b^			
IC lateral	Synthesis						0H (10)^b^			

Aud Ctx layer I	Synthesis						+1H (10)^b^			
Aud Ctx layer II	Synthesis						−7H (10)^b^			
Aud Ctx layer III	Synthesis						+2H (10)^b^			
Aud Ctx layer IV	Synthesis						−6H (10)^b^			
Aud Ctx layer V	Synthesis						+1H (10)^b^			
Aud Ctx layer VI	Synthesis						+3H (10)^b^			

*See **Box 1** for table notes*.

Acetylcholine synthetic capacity in the LSO and the ventral nucleus of the trapezoid body (VNTB) decreased after cochlear ablation, perhaps as a retrograde effect of olivocochlear terminal destruction (14), but it increased at 5 months after intense sound exposure. Since these two regions give rise to the cholinergic olivocochlear bundle (108) and olivo-CN connections that terminate in CN granular regions (109), the increased choline acetyltransferase activities in the LSO and VNTB at long times after intense sound exposure, together with the increased activities in the CN granular region, may suggest an upregulation of olivo-CN projections.

### Serotonin and norepinephrine

Relatively, little work has been published concerning changes in other neurotransmitter-related chemistry in the central auditory system after cochlear damage. Increases in serotonin and norepinephrine metabolites have been reported within 45 min after white noise exposure in rat caudal CN (DCN + PVCN) and Aud Ctx (110). For the serotonin metabolite, there was a 34% increase in the CN and 22% increase in the Aud Ctx. For the norepinephrine metabolite, there was a 121% increase in the CN, but no measurable change in the Aud Ctx. Since these changes occurred after very short times at 70 dB sound pressure level but not at 90 or 110 dB, they represent increased metabolism but not effects of cochlear damage from acoustic trauma.

The effects of salicylate on the serotonin levels in the central auditory system were measured by microdialysis (111). Within few hours after systemic administration of salicylate, serotonin levels increased by about 170% in both the IC and Aud Ctx of rats. Another study investigated the effects of acoustic trauma on the density of serotonergic fibers (estimated by measures of serotonergic fiber length density using immunohistochemistry for serotonin reuptake transporter) in the IC of mice after 3–16 weeks (112). Decreases of 17, 10, and 14% were reported in dorsal, lateral, and central regions of the IC on the affected (contralateral) side as compared to ipsilateral. Although the measured changes in serotonin chemistry in these studies were in opposite directions at very different times and after different interventions, both would lead to an imbalance of neurotransmitter chemistry in the central auditory system, which might be associated with tinnitus. There is some evidence that changes in serotonin neurotransmission might have a role in tinnitus (2, 113, 114).

## Discussion

### Clinical applications

The compiled results from available studies suggest that different types of cochlear damage may lead to different neurotransmitter-related chemical changes in the central auditory system, even though they all could result in tinnitus. The changes following ototoxic drug administration resemble those after cochlear ablation, although with a slower development and smaller magnitude, but the changes at long times after intense sound tend to be in the opposite direction. This implies that tinnitus may result from a variety of different, even contrasting, chemical changes. Further, the neurochemical changes reported for several different transmitter systems after cochlear damage suggest that imbalances involving any of the various transmitter systems could result in tinnitus. Perhaps, more detailed investigations would distinguish different types of tinnitus. It may also be useful to distinguish between tinnitus as a symptom, which commonly occurs acutely after peripheral insult or even spontaneously, and tinnitus as a chronic morbidity. Although it is reasonable to hypothesize that tinnitus may result from an increase in excitatory neurotransmitter chemistry and/or a decrease in inhibitory neurotransmitter chemistry, the available data suggest that the chemical basis for tinnitus may not depend on the direction of change in these chemistries so much as any imbalance between them. Another factor to consider is the magnitude of cochlear damage. Perhaps, the similarity of chemical effects after cochlear ablation and at longer times after carboplatin administration is related to the magnitude of inner hair cell loss, which has been proposed as the most important factor that leads to tinnitus (115). Cochlear ablation leads to total hair cell loss and carboplatin administration to almost total inner hair cell loss (11, 12), whereas intense tone exposure leads to more limited inner hair cell damage (73, 116). More detailed measurements of damage magnitudes may result in a clearer understanding of subsequent chemical changes.

A factor that confounds the application of results for animal experiments to human hearing loss and tinnitus is the variation in experimental results among animal species. The most consistent effect found was the decrease in glutamate and aspartate levels and glutamate release and uptake in the CN regions receiving major innervation from the auditory nerve following cochlear ablation. Even for this effect, however, if one only studied short post-damage survival times, the surprising increase of aspartate levels at these times in chinchillas would complicate interpretations of the data. Collection of data for a wide range of post-damage survival times showed that some apparent contradictions between the results for different species result from different time courses of the chemical changes after cochlear damage rather than major differences in the ultimate direction of the change.

### Need for more data

It is evident from the tables that most available neurotransmitter-related chemical data for the central auditory system after cochlear damage have been obtained for the CN and IC, and there is a relative lack of data for the MG and Aud Ctx. Further study at different levels of the central auditory system is needed. There is also a relative lack of data for effects of ototoxic drugs. Besides carboplatin, there are over 200 medications of several types that can adversely affect hearing (3). This underscores the need for more research on the effects of various drugs on the neurochemistry of the central auditory system.

### Future directions

The chemical effects of cochlear damage following intense sound or ototoxic drugs generally appear to develop more slowly than those following the more severe damage produced by surgical ablation of the cochlea. It may therefore be useful to expand the time frame for measuring chemical changes after intense sound or ototoxic drugs in order to identify changes that underlie chronic tinnitus. In the case of intense sound, the changes in amino acid levels found 5 months after the exposure were more consistent for several amino acids than those at times <2 months after exposure. The changes in choline acetyltransferase activity (for acetylcholine synthesis) in the CN remained significant only in the granular region at 5 months after exposure.

Some amino acids such as aspartate and taurine may be associated with tinnitus indirectly through metabolic or functional relationships to neurotransmitters. Additional studies on such amino acids may prove useful for understanding the neurochemical basis of tinnitus.

Perhaps because of various experimental limitations, such as insufficient sample size, small chemical changes in a given region may not reach statistical significance. However, patterns of change that are widespread over many regions might indicate functionally important neurochemical effects underlying tinnitus. Studies with larger sample sizes may improve the likelihood of showing statistically significant changes, but even data not reaching statistical significance may still contribute to the understanding of the chemical mechanisms associated with tinnitus.

The studies investigating the neurochemical changes underlying tinnitus comprise one component of a large body of tinnitus-related research. Progressive scientific advances in this component may contribute toward greatly needed improvements in the prevention, diagnosis, and management of tinnitus.

## Author Contributions

Both authors contributed to all aspects of this work.

## Conflict of Interest Statement

The Review Editor James A. Kaltenbach declares that, despite having collaborated with the author Donald A. Godfrey, the review process was handled objectively and no conflict of interest exists. The authors declare that the research was conducted in the absence of any commercial or financial relationships that could be construed as a potential conflict of interest.

## Abbreviations

AMPA, α-amino-3-hydroxy-5-methyl-4-isoxazolepropionic acid; Aud Ctx, auditory cortex; AVCN, anteroventral cochlear nucleus (generally more rostral portion); CN, cochlear nucleus; CN Granular, cochlear nucleus granular region, usually that portion adjacent to AVCN; CNQX, 5,6-cyano-7-nitro-quinoxaline-2,3-dione; DCN, dorsal cochlear nucleus, divided into deep layer and superficial layers (combined fusiform soma and molecular layers); GABA, γ-aminobutyric acid or γ-aminobutyrate; IC, inferior colliculus (IC ventral approximately corresponds to the central nucleus of the IC); LSO, lateral superior olivary nucleus; MG, medial geniculate; mRNA, messenger RNA (ribonucleic acid); NMDA, *N*-methyl-d-aspartate; PVCN, posteroventral cochlear nucleus (generally more caudal portion); VCN, ventral cochlear nucleus; VNTB, ventral nucleus of the trapezoid body.
